# Learning from a diabetes mHealth intervention in rural Bangladesh: what worked, what did not and what next?

**DOI:** 10.1080/17441692.2021.1923776

**Published:** 2021-05-08

**Authors:** Joanna Morrison, Kohenour Akter, Hannah Jennings, Naveed Ahmed, Sanjit Kumer Shaha, Abdul Kuddus, Tasmin Nahar, Carina King, Hassan Haghparast-Bidgoli, A. K. Azad Khan, Anthony Costello, Kishwar Azad, Edward Fottrell

**Affiliations:** a Institute for Global Health, University College London, London, UK; b Diabetic Association of Bangladesh, Dhaka, Bangladesh; c University of York; d Department of Global Public Health, Karolinska Institutet, Stockholm, Sweden

**Keywords:** mhealth, diabetes, non-communicable diseases, process evaluation

## Abstract

There is an urgent need for population-based interventions to slow the growth of the diabetes epidemic in low-and middle-income countries. We tested the effectiveness of a population-based mHealth voice messaging intervention for T2DM prevention and control in rural Bangladesh through a cluster randomised controlled trial. mHealth improved knowledge and awareness about T2DM but there was no detectable effect on T2DM occurrence. We conducted mixed-methods research to understand this result. Exposure to messages was limited by technological faults, high frequency of mobile phone number changes, message fatigue and (mis)perceptions that messages were only for those who had T2DM. Persistent social norms, habits and desires made behaviour change challenging, and participants felt they would be more motivated by group discussions than mHealth messaging alone. Engagement with mHealth messages for T2DM prevention and control can be increased by (1) sending identifiable messages from a trusted source (2) using participatory design of mHealth messages to inform modelling of behaviours and increase relevance to the general population (3) enabling interactive messaging. mHealth messaging is likely to be most successful if implemented as part of a multi-sectoral, multi-component approach to address T2DM and non-communicable disease risk factors.

## Introduction

Around 1 in 11 adults worldwide now have diabetes, of which 90% have type 2 diabetes (T2DM) which can largely be prevented by adopting healthier behaviours (Zheng et al., 2018). T2DM is the 7th biggest cause of death in low-and middle-income countries (LMICs) (WHO, 2018), with particularly high incidence in South Asia (Hills et al., 2018). The 2019 International Diabetes Federation Atlas estimates that the age adjusted comparative prevalence of diabetes in adults is 10.4% in India, 10.7% in Sri Lanka, 9.2% in Bangladesh, 10.3% in Bhutan, 19.9% in Pakistan, and 7.2% in Nepal (International Diabetes Federation, 2019). There is an urgent need to develop and test population-based interventions to reduce this disease burden.

Given the high penetration of mobile phones in LMIC contexts and their potential cost-effectiveness, mHealth may offer a scalable intervention to deliver educational advice, information and motivation for behaviour change (Gurman et al., 2012; Head et al., 2013). mHealth interventions targeting those with T2DM or at high risk of T2DM have shown some success at improving blood sugar control and reducing body mass index – a key risk factor for T2DM (Arambepola et al., 2016; Haider et al., 2019). mHealth has also been identified as a potentially cost-effective and feasible intervention to modify T2DM risk factors such as sedentary behaviors, obesity, unhealthy diets and low levels of physical activity in LMICs (World Health Organization, 2017). Systematic reviews have identified the need for rigorous evaluation and clear description of mHealth interventions to understand optimal delivery mechanisms, frequency, and message type (Gurman et al., 2012; Hall et al., 2015; Kitsiou et al., 2017).

We present findings from process evaluation research of a theory-based mHealth intervention implemented in rural Bangladesh and evaluated through a cluster randomised controlled trial(cRCT) (Fottrell et al., 2019). Using mixed methods, we describe the implementation of the intervention and explore how it was effective in increasing knowledge and awareness of T2DM but had no detectable effect on population-level occurrence of T2DM and intermediate hyperglycaemia. We then make recommendations about the implementation of population-level mHealth interventions to increase their potential impact on T2DM and other non-communicable diseases.

### Mhealth in Bangladesh

Out of a population of ∼165 million, and there are 163 million mobile phone users in Bangladesh (Bangladesh Telecommunication Regulatory Commission, 2020), and most households have a mobile phone (Bangladesh Bureau of Statistics, 2019). Whilst there are significant gender differences in ownership, with 86% of men owning phones, compared with 58% of women (Rowantree, 2019), recent survey data suggest that 98% of women aged 15–49 years had used a mobile phone during the last 3 months. Most internet usage is through mobile phones (Alliance for Affordable Internet & Programme, 2019) but only 24% of the population have a smart phone (LIRNEasia, 2019) and only 53% of households in urban areas, and 33% in rural areas have internet access (Bangladesh Bureau of Statistics, 2019).

#### mHealth for T2DM in Bangladesh

The 2011 Bangladesh Demographic and Health Survey reported that among those over 35, 24% had abnormal fasting glucose and 8% had diabetes (NIPORT, Mitra and associates, & ICF International, 2013). The prevalence of T2DM is predicted to rise to 23.6% (13.6–36.3) for men and 33.5% (19.9–50.9) for women by 2030 (Rahman et al., 2017). Given the population coverage of mobile phone use in Bangladesh, high levels of acceptability (Ahmed et al., 2014; Khatun et al., 2016), and potential for cost-effectiveness (Islam et al., 2020), mHealth interventions have may an important role in reducing the T2DM burden. mHealth interventions have had some success in improving glycemic control amongst diabetics in urban Dhaka (Islam et al., 2015; Yasmin et al., 2020). Population level interventions in rural areas to prevent and control T2DM have, so far, not been rigorously evaluated. Therefore, we developed and evaluated an mHealth voice-messaging intervention which was delivered across the general population of mobile phone owning households in Faridpur District in rural Bangladesh.

### The trial

The mHealth intervention was evaluated as part of the DMagic three-arm cRCT. The trial evaluated the effectiveness of (1) mHealth messaging, and (2) community groups working through a participatory learning and action (PLA) cycle compared with control areas. We evaluated the effect of these interventions on the prevalence of intermediate hyperglycaemia and T2DM and two-year cumulative incidence of T2DM among an intermediate hyperglycaemia cohort(Fottrell et al., 2016). We strengthened health services in all three trial arms by delivering training on T2DM screening and referral to village doctors and pharmacy owners which were often used by the population in our study area. An estimated 61% of our target population of adults aged 30 years and above reported receiving at least one mHealth message during the study period.

The PLA arm of the trial showed a combined absolute reduction in T2DM and intermediate hyperglycaemia prevalence of 20.7% (95% CI 14·6–26·7), and an 8.7% reduction (3·5–14·0) in the two-year cumulative incidence of T2DM among those with intermediate hyperglycaemia in the PLA versus control arm, and the intervention was highly cost-effective (Fottrell et al., 2019). Trial results showed that mHealth had large, significant impacts on population knowledge and awareness of T2DM and how to prevent it but there was no evidence of effect of mHealth on health behaviours or on the occurrence of intermediate hyperglycaemia and T2DM in the population as a whole.

### Theory of change

Our formative research showed a lack of knowledge about T2DM, risk factors, symptoms, and preventative measures, and a low level of awareness among many people with T2DM about their diabetic status in this setting (Fottrell et al., 2018b; Jennings et al., 2019). We used the COM-B behaviour change wheel and the Transtheoretical Domains Framework (TDF) as the theory base of our intervention (Cane et al., 2012; Michie et al., 2011). The COM-B framework focuses on the interaction between capability, opportunity and motivation to generate behaviour. Capability is defined as an individual’s psychological and physical capacity to engage in the activity concerned. Motivation is the processes that energise and direct behaviour and includes habitual processes, emotional responding and analytical decision-making. Opportunity is defined as all the factors that lie outside the individual that make the behaviour possible or prompt it. The different elements are interrelated. For example, opportunity can influence motivation, as can capability; engaging in a behaviour can alter capability, motivation and opportunity. The TDF framework identifies 14 overarching behavioural domains mapped according to capability, opportunity and motivation. Through this mapping and categorising of behaviour, different behaviour change techniques can be drawn on to encourage behaviour change.

Our hypotheses were that mHealth messages would: (1) educate, persuade, train, and model behaviours which would encourage our population to change their behaviours in relation to four T2DM risk factors: smoking, diet, stress and physical activity; (2) encourage earlier diagnosis and better control through improved care-seeking behaviour; (3) encourage interaction in communities about T2DM and risk factors which would enable messages to reach others besides the listener and help to develop an enabling environment for behaviour change. Our formative research enabled detailed mapping of context specific capability, motivation and opportunity related barriers and enablers within behavioural areas of smoking, stress, diet, physical activity and care-seeking (Jennings et al., 2019). We worked with scriptwriters and medical experts to develop accurate, engaging messages.

### The intervention

mHealth messages were available free of charge to anyone with access to a mobile phone in the intervention areas. People signed up to receive messages by giving their mobile phone number to community recruiters at the beginning of the intervention period or at three months into the intervention period. Initially there were 9381 recipients, and this dropped slightly to 8980 at the end of the intervention, after 14 months. 6259 (66.7%) recipients were men and 3122 (33.3%) were women. Recipients were given the two numbers that were likely to send the message and asked to save these. We sent two one-minute messages every week on Fridays and Mondays between 8-8.30pm. An automated system detected if the message was not received the first time, and it was re-sent on Saturday and Tuesday at 7pm.

**Figure 1.**
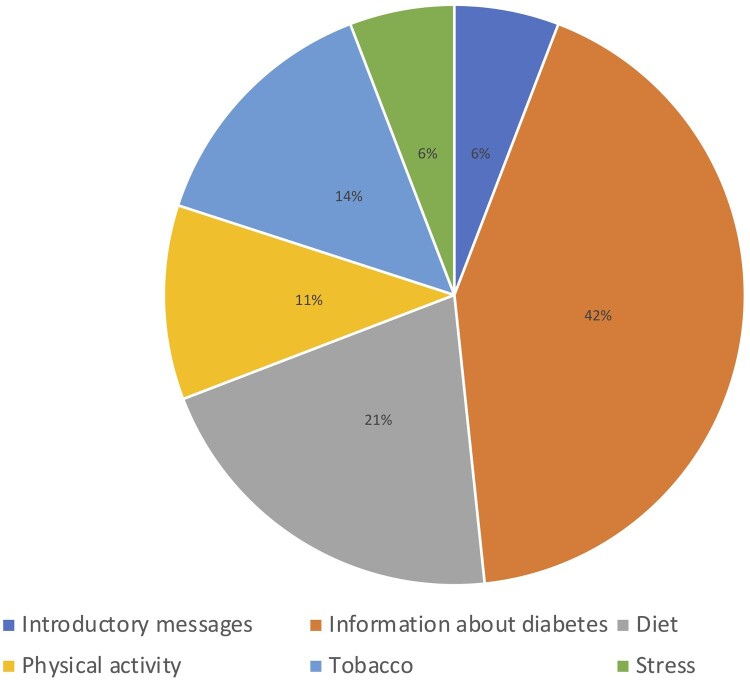

The first message introduced the sender, the Diabetic Association of Bangladesh (BADAS), and explained the importance of good health and prevention and control of T2DM. It also detailed the time and days when we would be sending messages and reminded recipients that they would not be charged for receiving messages. Recipients were also encouraged to share and discuss messages with friends and family members in this introductory message. This introduction message was sent a further six times throughout the intervention period. 120 messages were sent in total. Messages included information on signs, symptoms, prevention, and care for T2DM, and provided examples of strategies to reduce the risk of T2DM and its complications (n = 51). Messages about preventing T2DM focused on diet (n = 25), tobacco (n=17), physical activity (n = 13) and dealing with stress (n = 7) (Figure 1). During Ramadan, messages contained advice on fasting and T2DM management, regardless of the religion of the receiver.

Messages used five formats: drama (n = 60), information given by a doctor (n = 24), information given by an announcer (n = 20), song (n = 5), and narrative fictional personal experience (n = 4). Most (n = 67) messages sought to develop knowledge, or model behaviour (n = 46). Most messages that sought to develop knowledge were information given by a doctor (n = 24), and most of those which modelled behaviour were dramas (n = 35). Messages sought to reach a broad audience and therefore featured a mix of men and women of different ages. Of the 60 messages which used drama (including dramatisation of doctor patient interactions), 34 (56.7%) were interactions between men, 19 were between men and women (31.7%) and seven (11.7%) were between women.

## Methods

### Setting

The trial was implemented in four upazillas (Saltha, Boalmari, Nagarkanda and Madhukhali) of Faridpur District in central Bangladesh where we had established relationships with community stakeholders and local decision makers. Faridpur is typical of rural areas in Bangladesh, and has a population of over 1.7 million, with a mainly agricultural economy of jute and rice farming. Our baseline data from 2016, showed that the overall prevalence of intermediate hyperglycaemia and T2DM was 17.2% and 8.9% among men and 23.4% and 11.5% among women, respectively (Fottrell et al., 2018a). T2DM services were available at low-cost from the BADAS hospital in the urban District headquarters of Faridpur; but in rural areas remoteness and flooding affected access to services, and quality of health services was variable. The population was mainly Bengali and almost 90% were Muslim (Bangladesh Bureau of Statistics, 2013).

We conducted two surveys of recipients and non-recipients in December 2016 (after sending 16 messages), and in July 2017 (after sending 75 messages) to explore preferences and to describe the factors affecting receipt of messages. We collected qualitative data during and after the intervention had been implemented and we collected data from our server on the number of messages that were received, and the day of receipt.

### Surveys

#### Sampling

Given that data from surveys were used to inform the development of messages, we aimed for around a 1% sample of subscribers. As part of our cluster randomised controlled trial, we had conducted a household census of all individuals aged 30 years and above in all study villages to create a sampling frame. We used two-stage sampling to select individuals for this mHealth process evaluation survey. In stage one we randomly selected one village in each upazilla from the sampling frame using probability proportional to size. In stage two, we used simple random sampling to select mHealth recipients (from the list of recipients in that village) and non-recipients (from the sampling frame, with recipients removed). We called those selected and took verbal consent to participate, before a trained data collector from the local area visited them in their homes to administer a questionnaire. Recipient records did not disaggregate by gender, and our random sample for the first survey contained more men than women. Therefore, for the second survey we randomly selected one village in each upazilla from the sampling frame using probability proportional to size, and then used stratified random sampling to ensure equal numbers of men and women. We interviewed 109 recipients and 105 non-recipients.

#### Data collection, management and analysis

Two questionnaires were developed, one for recipients and one for non-recipients, and administered on mobile phones using Open Data Kit software. We developed options for closed questions on the basis of our non-participant observation data, pilot tested them on 12 men and eight women and adapted the questions and response options. We adapted a few questions in the second survey and piloted this with seven women and eight men. We collected socioeconomic and socio-demographic data and explored factors affecting receipt of messages, recall of messages, and preferences over type and content of messages. We conducted descriptive analysis stratified by gender and age in SPSS.

### Qualitative data collection

The mHealth coordinator visited intervention areas once a month, and the Process Evaluation (PE) Manager and the Monitoring and Evaluation (ME) Manager visited intervention areas six times. During field visits they discussed whether messages were being received and listened to, what affected receipt of messages, and what affected the decision to subscribe. The mHealth coordinator and ME Manager discussed their findings with the PE Manager who maintained field notes later summarised in PE reports throughout the intervention period. mHealth server data were also recorded in process evaluation reports. After the intervention had been completed, the PE Manager (a trained qualitative researcher) conducted four focus group discussions (FGDs) in three upazillas with a convenience sample of recipients, to explore how the intervention might have been effective at changing behaviour and explore the findings from the mHealth surveys. FGD participants may or may not have also answered survey questions. We conducted two FGDs with men and two with women. FGDs had a minimum of 7 and a maximum of 11 participants. We also conducted one key informant interview (KII) with a recipient in the fourth upazilla to discuss their experience and examine triangulation of FGD and observation data. Data were recorded and directly transcribed into English for analysis in Nvivo v11. We conducted descriptive content analysis (Green & Thorogood, 2005). The first author and the PE Manager read and made notes about the data. The first author developed and applied the initial coding framework, categorising text under inductive themes of shame, fear, gender, relevance, trust, self-blame, habit, desire, message fatigue, method of motivating, message timing and message content and wrote a description of the data. She discussed this with the PE manager, and merged some of the themes. The main themes were compared with the written reports of the quantitative data to construct a narrative explaining why the intervention may not have affected T2DM outcomes.

This research has been reviewed and approved by the University College London Research Ethics Committee (4766/002) and by the Ethical Review Committee of the Diabetic Association of Bangladesh (BADAS-ERC/EC/t5100246). The trial has been registered and assigned an International Standard Randomised Controlled Trial Number (ISRCTN41083256).

## Results

### Non recipients

Demographic characteristics of non-recipients and recipients in both surveys are presented in Table 1; the first survey had significant differences between recipients and non-recipients. Of the non-recipients, 60% and 63% did not have a personal mobile phone, across both surveys. Amongst those with a mobile phone, reasons for not receiving messages differed between the surveys, with not wanting to give their number being the most common reason in the first survey (53%), and a family restriction in the second (69%) – Table 2. The first survey showed that 11 (11.5%) non-recipients had heard a message that was shared by a friend or family member, which increased to 45 (42.9%) in the second survey.

**Table 1.** Recipient and non-recipient demographic characteristics.

Demographic information	First survey			Second survey		
	Recipients N (%)	Non-recipients N (%)	*p*-value	Recipients N (%)	Non-recipients N (%)	*p*-value
**Sex**			0.039			0.887
Female	49 (45.4)	30 (31.3)		54 (49.5)	51 (48.6)	
Male	59 (54.6)	66 (68.8)		55 (50.5)	54 (51.4)	
**Person who knows they have diabetes**	N/A	N/A		9 (8.3)	9 (8.6)	0.934
**Age**			<0.001*			0.667
18–29	13 (12.0)	0		N/A	N/A	
30–39	31 (28.7)	13(13.5)		20 (18.4)	16 (15.2)	
40–49	27 (25.0)	22 (22.9)		15 (13.8)	17 (16.2)	
50–59	16 (14.8)	22 (22.9)		22 (20.2)	19 (18.1)	
60–69	12 (11.1)	23 (24.0)		32 (29.4)	26 (24.8)	
70+	9 (8.3)	16 (16.7)		20 (18.4)	27 (25.7)	
**Currently married**	97 (89.8)	76 (70.2)	0.034	73 (67.0)	61 (58.1)	0.180
**Highest level of education**			<0.001			0.497
No formal	45 (41.7)	65 (67.7)		60 (55.1)	64 (61.0)	
Incomplete primary	29 (26.9)	20 (20.8)		27 (24.8)	26 (24.8)	
Completed primary or above	34 (31.5)	11 (11.5)		22 (20.2)	15 (14.3)	
**Occupation**			0.064*			0.790*
Unemployed (inc housework, remittance and unable to work)	51 (47.2)	33 (34.4)		61 (56)	63 (60)	
Manual (inc home-based work)	50 (46.3)	60 (62.5)		44 (40.4)	39 (37)	
Professional (inc village dr)	7 (6.5)	3 (3.1)		4 (3.7)	3 (2.9)	
**Total**	**108**	**96**	** **	**109**	**105**	** **

*Fishers exact test used.

**Table 2:** Non recipient exposure to messages and reason for not receiving messages.

	First survey N = 96 (%)	Second survey N = 105 (%)
**Have a mobile phone**	38 (39.6)	39 (37)
**Why don’t you receive messages?**		
Didn’t want give the number	20 (52.6)	11 (28.2)
Family restriction	1 (2.6)	27 (69.2)
I was not at home during subscription	9 (23.7)	-
I have a new mobile number	6 (15.8)	-
Other	2 (5.3)	1 (2.6)
**Someone in my family receives messages**	**11** (**11.5)**	**32**(**31)**
**My neighbours receive the messages**	**31** (**32.3)**	**26**(**25)**
**Someone has shared the messages with me**	**11** (**11.5)**	**45** (**43)**
family member	3 (27.3)	18 (40)
relative	2 (18.2)	3 (6)
neighbour	6 (54.6)	24 (53)
**Have changed behaviour as a result of the messages**	**1** (**9.1)**	**24** (**53)**
**What changes?**		
Doing exercise	1 (100)	23 (96)
Eating more vegetables and fruit	1 (100)	20 (83)
Avoid smoking		1 (4.2)

### Message exposure among recipients

**Figure 2.**
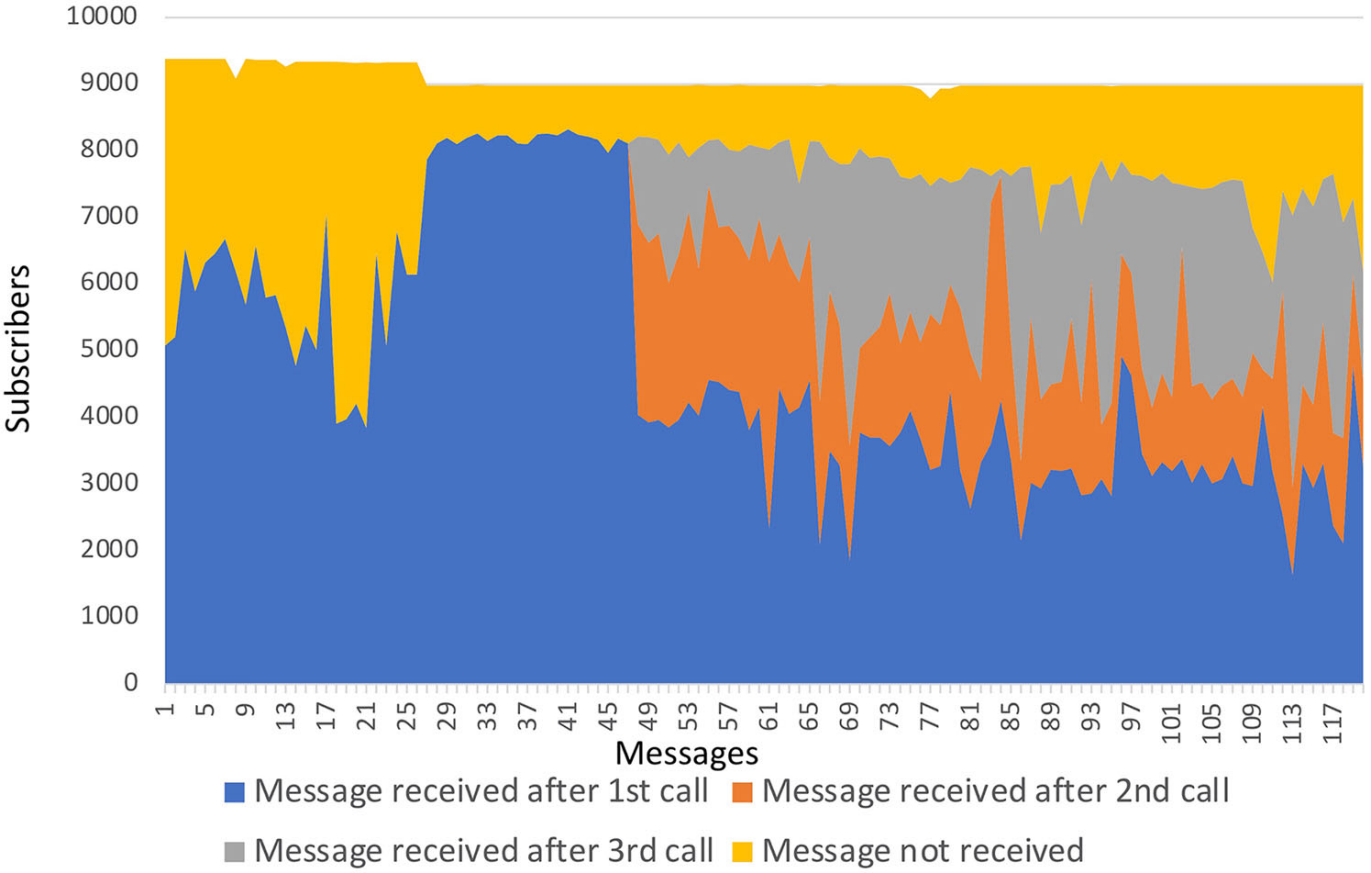

Data on whether the message had been received were available from the 17th week of the intervention onwards. From the 17th until the 27th week, there was a relatively low rate of message receipt, with only 42% of recipients receiving the 18th and 21st message. The ME Manager called those numbers who were consistently not receiving messages to check if they were in-service. 1102 numbers were deleted, and a new server was installed. 754 new recipients were recruited after the first mHealth survey. In order to increase exposure to messages, we also increased the number of times we re-sent messages from two times to three times. Just over half (n = 62) of the messages were received on the first call (Figure 2).

In the first survey, 64 (66%) of respondents reported that they had stopped listening to at least one of the messages and 31 (32.3%) of respondents reported that they had stopped listening to messages either frequently or sometimes. In the second survey, half of respondents (n = 55) reported having stopped listening to messages before they were completed. 21 (19.3%) respondents reported stopping listening to messages either frequently or sometimes. The most common reason for disconnection was because the respondent was busy with other activities (Table 3).

**Table 3.** Engagement with messages.

	First survey N = 96(100%)	Second survey N = 109(100%)
**Stopped listening to messages part of the way through**	** **	** **
Never	32(33.3)	54(49.5)
Frequently (more than half)	10(10.4)	8(7.3)
Sometimes (around half)	21(21.9)	13(11.9)
Occasionally (one or two)	33(34.4)	34(31.2)
**Reason stopped listening**	**63** (**100)**	**55** (**100)**
Busy with other things	49(76.6)	42(76.4)
Not interested	14(21.9)	15(27.3)
Did not recognise the message	8(12.5)	6(10.9)
Already heard the message	-	5(9.1)
Other reason	4(6.3)	9(16.4)

### Message format preferences of recipients

Amongst those receiving messages, a preference for the messages that were from a doctor was reported in both surveys (74% and 71%). This was triangulated in the qualitative data as these were easy to understand, were trusted and carried authority. In the second survey more recipients preferred dramas (22% vs 13%), but preferences were mostly similar by age and gender. In FGDs, men felt the dramas were confusing for those with low literacy and that the key message was lost. Data from both surveys indicated that most people understood the messages. There was an increase from 88.5% (85) recipients in the first survey saying that the message was always interesting or enjoyable to 93.6% (102) recipients in the second survey. This could be because we adjusted the messages after the first survey to feature more women and to avoid consecutive messages on similar topics.

### Factors affecting exposure to messages

#### Message fatigue

Participants suggested that messages might be rejected because they were assumed to be advertising, commercial entertainment offers, social networking or other unsolicited messaging: ‘(People) cannot differentiate between the unsolicited messages and the messages giving health advice. Most of the time, the health messages are being avoided because people don’t know the difference’ (KII). Men and women were irritated with frequent unsolicited messaging and a few worried it was using up their phone credit: ‘I have scolded the customer care officials often to stop such unnecessary unsolicited messages that we are sometimes charged for’ (Men FGD 026). Some indicated that they would have listened more frequently to the messages if they had known they were from BADAS: ‘I rejected many times … I thought they were all fraud … now we understand that those messages were important’ (Men FGD 026).

#### Relevance

Those with T2DM and participants whose family members had T2DM found the messages relevant to them: ‘At first, some of my relatives treated unsolicited messages and health messages in same way. But they started following the messages from BADAS after being affected by diabetes’ (KII). But many participants reported that the messages were only relevant for those affected by T2DM. One man told us: ‘I have never checked and am not diabetic, so there is no benefit in listening to those messages’ (Men FGD 026). Men and women felt that many community members did not feel at risk of T2DM, which affected their interest in the messages: ‘Some people receive messages but do not listen to them. They are totally not interested to listen … People who are energetic, or not prone to diseases they might not care (about the messages)’ (Women FGD 023). A group of men told us: ‘Most people are less interested (in changing their behaviour) unless they are obliged to. People here do not give importance (to their health) until they get sick’ (Men FGD 025).

#### Diffusion of messages

We asked if participants discussed the messages or listened to them with anyone else and findings were slightly different in each survey. The first survey showed that 53 (55.2%) recipients shared at least one message and 36 (37.5%) recipients shared at least half of the messages they had received. 65%(n = 34) of men shared messages compared with 37% (n = 17) of women and messages were shared more with friends and neighbours (n = 37, 72.6%) than with family members (n = 30, 58.8%). In the second survey, 50 recipients (46.9%) reported sharing at least one message and 35 recipients (32%) reported sharing at least half of the messages they had received. In qualitative data women reported hearing the messages from their husbands and sons’ phones, and men reported group listening: ‘After sunset, we sit in the tea stall all together and listen to the message. I always kept my loud-speaker on … Yes we listened at his shop and discussed about messages. They are talking about walking and giving such good advice. We always appreciated and discussed … but on reaching home, we forgot everything’ (Men FGD 026).

### Factors affecting behaviour change

#### Motivation

In the first and second quantitative surveys, 50 (52%) and 56 (53%) recipients reported behaviour change since the mHealth messaging, all related to diet and physical activity, except one respondent who reported avoiding smoking. During qualitative data collection, before analysis of the trial results, we discussed whether participants thought that the incidence of T2DM would decrease as a result of the mHealth messages. Whilst many participants reported that the messages developed their knowledge about T2DM, most felt that the message alone was not sufficient to motivate behaviour change.

Habits and desires were difficult to address through mHealth messaging. Women said: ‘The mobile messages regularly instruct us how to cook in a healthy way, what should be eaten, and to exercise … but we have not paid attention to those as we have our own habits’ (Women FGD 024). A group of women discussed their desire for sweet food, and the expectation to overeat at social occasions: ‘I could not give it up, I have an immense desire to have sweets or sweet food … if we are invited anywhere we eat so much … I cannot resist, we must eat a lot there’ (Women FGD 023). This enjoyment of food and fear of a change in dietary behaviours prevented some men and women from testing for T2DM: ‘I’m so afraid to go for a test in case I get diagnosed as having diabetes … .After diagnosis you cannot eat sweets, you have to take *ruti* (bread). I do not enjoy having *ruti* … It’s an additional tension if anyone gets diagnosed with diabetes’ (Women FGD 024).

Participants described themselves and others as ‘careless’ (*udasin*) and acting with disregard for their own health. The key informant told us that ‘unconsciousness and carelessness are the main reasons behind the unhealthy lifestyle in our society.’ Both men and women cited laziness as a reason for not doing more physical activity, not having a T2DM test, and generally not following the messages: ‘We are being updated about diseases like diabetes. But we cannot behave according to the instruction because of our idleness’ (Women FGD 024).

Men and women suggested that they would feel more motivated and learn more easily and effectively through group discussion than through messages. This information was volunteered without knowledge of the PLA group approach. ‘In person’ discussions were considered enjoyable, would help recall and be more influential: ‘When we got a message, we discussed it amongst our family or friends. Some changes were made, but we could not maintain many of the rules due to our carelessness. We also couldn’t hear the message several times. Some people did not pay attention to the message. Your physical presence is more important. For example, you are here talking with us and we can easily recall, and we pay more attention’ (Men FGD 025). ‘If anyone discussed with us directly like how we have discussed today then it would be more effective … We enjoyed this discussion and we think it is more effective than messages. We get conscious (*sacheton*) and it increases our desire for information and desire to follow the advice more closely than if we just received messages’ (Women FGD 023). Participants felt that another benefit of sharing information in a group was to motivate more people to change their behaviour: ‘Except phone messages, we can be more aware about healthy lifestyle through physical meeting. This kind of group discussion should be conducted regularly. Some of us who are conscious (*sacheton*) can share our experience and knowledge with others’ (Men FGD 025).

#### Enabling environment

We discussed factors which prevent changes in behaviour, and many of these were related to maintaining status and social norms. For example, men told us that they felt uncomfortable buying cheap, plentiful healthy food: ‘I do not buy spinach which I can get at the market because it’s an issue of prestige. If I buy spinach from the market people might criticise me’ (Men FGD 026). Men also discussed being embarrassed about behaving in ways that suggested that they have T2DM, such as going for a morning walk or changing their diet: ‘I started to walk after listening to the messages then on the first day my friend asked me whether I was diabetic and I stopped walking … I felt embarrassed as people will think I’m diabetic … yes … .we do not go for walk as people think we are diabetic … ’ (Men FGD 026). Another man with T2DM told us: ‘After I was diagnosed with diabetes, I gave up sugar but I did not tell anyone as I felt embarrassed. One day I went to a tea shop and he had put so much sugar in the tea that I could not drink it, but even then, I could not say anything’ (Men FGD 025).

Women and some men discussed the effect of gender norms in preventing them from improving their diet, increasing physical activity or care-seeking for T2DM. Expectations that women should stay at home and be responsible for housework affected women’s ability to act on messages. Women discussed the possibility of going for a morning walk: ‘You need to provide training to men … walking is possible if they want us to walk … .If we talk to them now about walking then they will scold us’ (Women FGD 024). Some women depended on male accompaniment to leave the house for T2DM screening or check-ups and found it difficult to make dietary changes without the support of their husband and children: ‘I am diabetic and I have heard the messages that are similar to the doctor’s advice. Though I want to change some issues, I cannot … She [indicating to another group member] cannot reduce consuming oil even if she wants to because she has a husband and children’ (Women FGD 024). Men reported not wanting to ‘battle with our wives’ over dietary changes (Men FGD 026). One participant felt there needed to be improvements in services as well as increased awareness.

## Discussion

mHealth messaging increased knowledge and awareness about T2DM in communities that had received messages, and findings show that messages were discussed and disseminated. Message content was informed by detailed formative research, but exposure to messages was not optimal due to technological faults at the beginning of the intervention, a high frequency of mobile phone number changes, message fatigue and perceptions that messages were most relevant to those who had T2DM. Persistent social norms, habits and desires made behaviour change challenging, and participants suggested that they would be more motivated by group discussions than mHealth messaging alone. Our qualitative and quantitative samples were small but appropriate to inform the development of messages and understand their effect. There was a high degree of triangulation among different respondents, and from data collected through a variety of methods. We discuss the implications of our findings for the design of population level mHealth messaging interventions.

### Increasing exposure through active recruitment and trust

We recruited mobile phone owners in their homes and communities through face-to-face contact. It was important to have systems in place to check and update phone numbers in our database and have an active and ongoing recruitment process to deal with the frequent changes in phone numbers in our population. This issue was also identified in a recent review of implementation challenges of mHealth interventions, and the need for robust management information systems to analyse implementation, response and effect was also emphasised (van Olmen et al., 2020).

Unsolicited messaging was common in this context and could eliminate the need for ongoing recruitment, but participants in our study reported being tired or irritated with commercial messages and found it difficult to distinguish mHealth messages from these. Whilst it helped to alert recipients to the numbers that would be sending the messages, this would not be possible at scale. If using unsolicited messaging across populations, mHealth messages should clearly distinguish themselves to increase receipt. This study and other research has shown that the trustworthiness of the source of the mHealth intervention influences receptiveness and response (Peprah et al., 2020; Steinman et al., 2020). We found that the reputation and credibility of BADAS as a trusted source of health information about T2DM helped engagement with the messages. Future interventions need to research the extent to which the delivering organisation is perceived to be a trustworthy source of information.

### Increasing participant engagement with messages

It is of interest that while some participants enjoyed the story-based approach, others found it trivial. Allowing participants choice in the type of message they received may be beneficial in future interventions, or using multiple media (e.g. print, TV, radio) which would allow for different formats to respond to different preferences and increase opportunities for exposure (Wakefield et al., 2010). The simple delivery of messages allows for low literacy populations to be reached (Khatun et al., 2016), but a more engaged, participant-led messaging system might increase exposure to messages. Interactive approaches have been identified as more successful than one way communication of information in reviews of mHealth interventions with high-risk groups(Free et al., 2013; Watkins et al., 2018). It may be challenging to implement this at a population level and at scale, particularly given the lack of smart phone ownership among our population, but innovation in this area is possible.

### Increasing participant engagement in message design

Social modelling of behaviours by those who were at risk of T2DM or had T2DM were used in many of our messages. The literature suggests that modeling is increased when individuals want to affiliate with the model, or perceive themselves to be similar to the model (Cruwys et al., 2015). Our messages might have had less impact on people without T2DM, as they did not see themselves, or a person they would like to be, in the message. We designed the messages according to how we perceived participants (at risk of T2DM) instead of designing messages around how participants saw themselves (not at risk). Integrating participatory action research to the intervention could help to increase the relevance of the messages and ensure they respond to participant priorities and interests (Chhoun et al., 2019; Toefy et al., 2016).

### Addressing opportunity and motivation

Our intervention focused on making changes in the capability domain of COM-B (Michie et al., 2011), and was successful in increasing knowledge about T2DM in mHealth intervention areas. Whilst improved knowledge is an important part of T2DM prevention and control, stimulating changes in the enabling environment (opportunity domain) and the motivation domain is also required. Whilst messages might have increased awareness and discussion of harmful social norms and habits, structural constraints such as those related to gender (Morrison, Jennings, et al., 2019), inadequate access to quality T2DM screening and care (Lewis & Newell, 2014), and poor regulation of the commercial determinants of T2DM (Buse et al., 2017) could not be addressed in a 14-month mHealth intervention. Habits, desire, addiction, a need to conform to social norms and not feeling at risk of T2DM curtailed motivation for behaviour change in our population. Recipients were keen for face-to-face interventions and group-based approaches can help to create an enabling environment for behaviour change (Morrison, Akter, et al., 2019).

Our population level intervention was similar to mass media approaches to behaviour change. Multiple systematic reviews have failed to show conclusive effects of mass media on habitual and ongoing behaviours such as smoking (Bala et al., 2017; Carson-Chahhoud et al., 2017), non-communicable diseases (Mosdøl et al., 2017), health services utilisation (Grilli et al., 2002), and other health behaviours (Stead et al., 2019), but might be more effective if targeting a one-off behaviour, such as a vaccination campaign (Victora et al., 2016). mHealth interventions at a population level have value as an integral part of multi-sectoral action plans with multi-component interventions to tackle T2DM and other non-communicable diseases (World Health Organization, 2017) but may be of less use as a stand-alone intervention for complex, habitual behaviours with multiple social determinants.

## Conclusion

Rigorous mixed methods evaluation of population level mHealth interventions which measure their effect on health outcomes are rare but important in developing evidence about how to prevent and control T2DM and other non-communicable diseases. Our cRCT in a rural area of Bangladesh showed that mHealth messaging increased knowledge and awareness of T2DM but had no detectable effect on the population prevalence of T2DM and intermediate hyperglycaemia. We suggest that exposure to and engagement with messages can be optimised by (1) sending identifiable messages from a trusted source (2) increasing population participation in the design of mHealth interventions to inform modelling and increase relevance to T2DM and non-diabetics (3) participant interaction with an mHealth message to enable choice according to preferences. We suggest that mHealth messaging should be implemented as part of a multi-component multi-sectoral approach to motivate and empower communities and create an enabling environment for behaviour change.
